# *miR-9a* regulates levels of both *rhomboid* mRNA and protein in the early *Drosophila melanogaster* embryo

**DOI:** 10.1093/g3journal/jkac026

**Published:** 2022-02-10

**Authors:** Lorenzo Gallicchio, Sam Griffiths-Jones, Matthew Ronshaugen

**Affiliations:** 1 School of Biological Sciences, Faculty of Medicine, Biology and Health, Michael Smith Building, The University of Manchester, Manchester M13 9GB, UK; 2 School of Medical Sciences, Faculty of Medicine, Biology and Health, Michael Smith Building, The University of Manchester, Manchester M13 9GB, UK

**Keywords:** *miR-9a*, *rhomboid*, embryogenesis, single-cell quantification, smFISH

## Abstract

MicroRNAs can have subtle and combinatorial effects on the levels of the targets and pathways they act on. Studying the consequences of a single microRNA knockout often proves difficult as many such knockouts exhibit phenotypes only under stress conditions. This has often led to the hypothesis that microRNAs buffer the effects of intrinsic and environmental stochasticity on gene expression. Observing and understanding this buffering effect entails quantitative analysis of microRNA and target expression in single cells. To this end, we have employed single-molecule fluorescence in situ hybridization, immunofluorescence, and high-resolution confocal microscopy to investigate the effects of *miR-9a* loss on the expression of the serine-protease Rhomboid in *Drosophila melanogaster* early embryos. Our single-cell quantitative approach shows that spatially, the *rhomboid* mRNA pattern is identical in WT and *miR-9a* knockout embryos. However, we find that the number of mRNA molecules per cell is higher when *miR-9a* is absent, and the level and temporal accumulation of rhomboid protein shows a more dramatic increase in the *miR-9a* knockout. Specifically, we see accumulation of rhomboid protein in *miR-9a* mutants by stage 5, much earlier than in WT. The data, therefore, show that *miR-9a* functions in the regulation of *rhomboid* mRNA and protein levels. While further work is required to establish whether *rhomboid* is a direct target of *miR-9* in *Drosophila*, our results further establish the *miR-9* family microRNAs as conserved regulators of timing in neurogenic processes. This study shows the power of single-cell quantification as an experimental tool to study phenotypic consequences of microRNA mis-regulation.

## Introduction 

*Drosophila melanogaster* embryonic, larval, and adult development has provided an extremely important model for the study of microRNA (miRNA) biogenesis and function (Matranga *et al.* 2005; Rand *et al.* 2005; Okamura *et al.* 2007). MicroRNAs are short ∼22 nucleotide, single-stranded, endogenous RNAs found in animals and plants (Bartel 2004; Kozomara *et al.* 2019). MicroRNAs regulate gene expression post-transcriptionally by recruiting the RNA-induced silencing complex (RISC) and then binding to specific sequences on target mRNA molecules, usually in their 3’UTR. The binding of the miRISC triggers repression of translation, deadenylation, and/or degradation of the target mRNA (Valencia-Sanchez *et al.* 2006). It is estimated that the majority of animal mRNAs are targeted by miRNAs (Friedman *et al.* 2009; Agarwal *et al.* 2015). An intriguing dichotomy regarding the phenotypic consequences of miRNA mis-regulation has arisen, with gain of function (GOF) and loss of function (LOF) studies in different organisms suggesting different functional modes: LOF studies find that miRNAs are minor modulators, whereas GOF studies reveal them to be key regulators of gene expression (Reinhart *et al.* 2000; Miska *et al.* 2007; Alvarez-Saavedra and Horvitz 2010; Chen *et al.* 2014).

In many cases, individual effects of miRNAs on the expression of a target are relatively small (Miska *et al.* 2007; Alvarez-Saavedra and Horvitz 2010; Chen *et al.* 2019a). In addition, each miRNA may target hundreds of different transcripts, and many different miRNAs have been found to act on the same targets (Peter 2010). It is therefore expected that a high degree of quantitative precision is required to determine the specific effects of miRNAs on gene expression. Indeed, a complete understanding of miRNA function will only come from a precise quantitative analysis of miRNA activity at the single-cell level. Single-cell studies of miRNA effects on gene regulation may provide insight into cellular phenotypes that are not apparent at a tissue or organism level (Miska *et al.* 2007; Alvarez-Saavedra and Horvitz 2010). It has also been observed that the phenotypic effects of miRNA mutation or mis-regulation are sometimes only revealed under specific, often stressful, conditions (e.g. dietary restriction, temperature stress) (Li *et al.* 2009; Kennell *et al.* 2012). For example, flies lacking *miR-14* are more sensible to salt stress compared to WT, while flies lacking *miR-7* present abnormal expression of the proteins Yan and Ato only under temperature fluctuations (Xu *et al.* 2003; Li *et al.* 2009). Such stress-dependent miRNA phenotypes have also been observed in other organisms such as mouse and zebrafish (Van Rooij *et al.* 2007; Flynt *et al.* 2009). Thus, the phenotypic consequences of miRNA mis-regulation may be subtle and cryptic until particular environmental conditions expose the cellular level dysfunction.

The *mir-9* miRNA family is highly conserved in bilaterians and is a good example of a miRNA that can exhibit both subtle and strong phenotypes (Coolen *et al.* 2013). Experiments in a variety of vertebrate models show conservation of *mir-9* expression and function in neurogenesis and neuronal progenitor proliferation. Over-expression of *mir-9* in zebrafish embryos (Leucht *et al.* 2008), mouse embryonic cortex (Zhao *et al.* 2009), and chicken spinal cord (Otaegi *et al.* 2011) leads to a reduction of the number of proliferating progenitors, similarly to the observed effects in *Drosophila* (Li *et al.* 2006). However, when *miR-9a* was knocked out in *Drosophila*, the phenotype was quite mild, leading to a modest increase of sensory organ progenitors (SOPs) as well as some subtle wing-defects that were dependent on the genetic background (Li *et al.* 2006; Bejarano *et al.* 2010; Coolen *et al.* 2013). The overall complexity of miRNA-target genes networks and the observation that miRNAs do not generally have large effects on the levels of individual target genes lead to a model suggesting that many miRNA functions not as biological switches but rather as modulators or buffers of gene expression by fine-tuning the response to intrinsic and extrinsic noise (Liufu *et al.* 2017).

*miR-9* dysfunction has been associated with a number of human pathologies, including various kinds of cancer and neurodegenerative disorders (Coolen *et al.* 2013; He *et al.* 2017; Chen *et al.* 2019b; Khafaei *et al.* 2019). In medulloblastomas (a pediatric brain cancer) tumor cells appear to have decreased expression of *miR-9*, while in a subclass of glioblastoma (an aggressive adult brain cancer) tumor cells express *miR-9* at a higher level (Ferretti *et al.* 2009; Kim *et al.* 2011). *miR-9* has been found to have a role also in cancers not directly related to the nervous system, in which it may act as an oncogene or a tumor suppressor (Coolen *et al.* 2013).

The conserved role *miR-9* plays in the regulation of enhancer of split-HLH/HES family gene function in vertebrates and invertebrates strongly suggests an important ancestral function of *miR-9* (Bonev *et al.* 2011, 2012; Coolen *et al.* 2012; Soto *et al.* 2020). Work across a range of model organisms, including a number of studies in *Drosophila*, have focused on *miR-9a* as a modulator of the specification of *Drosophila* SOPs, a key neuronal cell type that emerges around embryonic stage 10 (Li *et al.* 2006; Cassidy *et al.* 2013). At embryonic stage 5, *miR-9a* is expressed in the dorsal ectoderm and neurogenic ectoderm: the germ layer where the future neuronal precursor cells will form (Fu *et al.* 2014; Gallicchio *et al.* 2021). This early expression throughout the neuroectoderm is reminiscent of early *miR-1* expression throughout the mesoderm, where *miR-1* functions in muscle development (Sokol and Ambros 2005). It has been suggested that both miRNAs likely respond to the Dorsal transcription factor (TF) gradient that activates and inhibits expression of genes involved in establishing the germ layers (Biemar *et al.* 2006). It is reported that *miR-9a* knock out (KO) flies show defects on the wing margin (Li *et al.* 2006) and an homozygous KO for *miR-1* causes lethality in second instar larvae, which die immobilized and with abnormal musculature (Sokol and Ambros 2005). When *miR-1* and *miR-9a* are mutated together, dramatic effects on embryonic development are observed (Fu *et al.* 2014). The double KO exhibits a disrupted pattern of *rhomboid* (*rho*) expression and a failure of gastrulation (Fu *et al.* 2014). *Drosophila rho* encodes a transmembrane serine protease (Rho), localized in the Golgi apparatus, that processes the epidermal growth factor (EGF) ligand Spitz, and is therefore necessary for proper EGF signaling (Bang and Kintner 2000;Urban *et al.* 2001). The pattern of *rho* expression is determined by dl activation and snail repression inputs (Bier *et al.* 1990). *rho* also has two predicted *miR-9a* binding sites in its 3’UTR. Together these observations suggest a role of *miR-9a* as a direct or indirect regulator of *rho* mRNA expression and/or translation.

We were therefore motivated to study *rho* expression and cellular phenotype in *miR*-*9a* mutant embryos at the single-cell level. In particular, single-cell quantitative approaches may reveal phenotypic consequences of relatively mild effects of miRNA mutations on gene expression levels, which might be lost when a population of cells is considered (Linsen *et al.* 2008). Using high-resolution confocal microscopy coupled with multiplex smiFISH and IF we examined expression domains, transcription dynamics and protein accumulation at the single-cell level in whole-mount developing *D. melanogaster* embryos. In *miR-9a* KO mutants, we observed an increase in both *rho* mRNA number per cell and Rho protein expression, concluding that *miR-9a* deletion affects *rhomboid* mRNA expression and protein accumulation. Together, these results show that single-cell analysis and quantification is a powerful approach to study miRNA function on target gene expression.

## Materials and methods

### Fly stocks, embryo collection, and fixing and larval dissection

Flies were grown at 25 or 18°C. Embryos were collected after ∼20 h and fixed in 1 V heptane + 1 V 4% formaldehyde for 30 min shaking at 220 rpm. The embryos were then washed and shaken vigorously for 1 min in 100% methanol. Fixed embryos were stored in methanol at −20°C. Larvae were dissected in 1× PBS, carcasses were fixed in 1 V 1× PBS + 1 V 10% formaldehyde for ∼1 h, washed with methanol, and stored in methanol at −20°C. Genotypes used for this study are: W [1118], (from Bloomington Drosophila Resource Centre) and *miR-9a*^E39^ mutants (Li *et al.* 2006) generously gifted by the Fen-Biao Gao lab.

### Probe design, smFISH, and immunofluorescence

We applied an inexpensive version (Tsanov *et al.* 2016; Morales-Polanco *et al.* 2021) of the conventional smFISH protocol in *Drosophila* (Trcek *et al.* 2017). Primary probes were designed against the mature *rho* mRNA (*rhomboid_e*), the first *rho* intron (*rhomboid_i*) and a genomic region flanking the *mir-9a* gene locus using the Biosearch Technologies Stellaris probe Designer (version 4.2). All sequences were obtained from FlyBase. To the 5′ end of each probe was added the Flap sequence CCTCCTAAGTTTCGAGCTGGACTCAGTG. Multiple secondary probes that are complementary to the Flap sequence were tagged with fluorophores (CAL Fluor Orange 560, CAL Fluor Red 610, Quasar 670) to allow multiplexing. Probes sequences are reported in the Supplementary Tables 1–3. For Immunofluorescence we used the following antibodies: mouse anti-Dorsal (Developmental Studies Hybridoma Bank #AB_528204) at 1:100, mouse anti-Spectrin (Developmental Studies Hybridoma Bank #AB_520473) at 1:100, guinea-pig anti-Rho gently gifted from the Hayashi lab at 1:400 (Ogura *et al.* 2018), goat anti-guinea pig IgG (H + L) Highly Cross-Adsorbed Secondary Antibody Alexa Fluor 555 (Invitrogen #A21435) at 1:500, and goat anti-mouse IgG (H + L) Highly Cross-Adsorbed Secondary Antibody Alexa Fluor 488 (Invitrogen #A32723) at 1:500.

### Imaging and quantification

Imaging was performed using a Leica SP8 Inverted Tandem Head confocal microscope with LAS X v.3.5.1.18803 software (University of Manchester Bioimaging facility), using 40×, and 100× magnifications. Deconvolution was performed using Huygens Pro v16.05 software. Membrane segmentation was performed on Imaris (version 9.5.0), mRNA molecules and Transcription sites were counted after membrane segmentation on Imaris 9.5.0 using the Cell module. Protein fluorescence levels were measured using FIJI for Macintosh. From each picture, five measurements of background mean intensity were taken. Each single measurement was then adjusted using the formula: integrated density—(area × background mean).

## Results

### *rho* and *mir-9a* are co-expressed in the neurogenic ectoderm

**Fig. 1.**
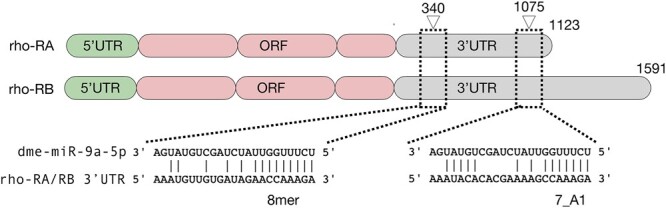
Binding sites of *mir-9a* on *rhomboid* 3’UTR. Schematic representation of the *Drosophila melanogaster miR-9a* binding sites on *rhomboid* 3’UTR. ORF, open reading frame. Numbers above 3’UTR region indicates nucleotide position from the 3’UTR start (indicated with number 0). Only the canonical microRNA binding site types have been considered.

Following the discovery of Rhomboid (Rho) as an intramembrane serine protease in *Drosophila*, Rho-like proteins have subsequently been identified in nearly every metazoan, (Urban *et al.* 2001; Freeman 2014). Although the molecular and cellular function of Rho-like proteins is well established, there are still a number of questions about their expression and possible post-transcriptional regulation. Given the strong *rho* phenotype exhibited in the *miR-1-miR-9a* double mutant we decided to investigate if *miR-9a* and/or *miR-1* could directly regulate *rho* mRNA degradation and/or translation. As *miR-1* is exclusively expressed in the mesoderm (Sokol and Ambros 2005; Fu *et al.* 2014) and *miR-9a* in the dorsal and neurogenic ectoderm (Fu *et al.* 2014; Gallicchio *et al.* 2021) largely overlapping *rho* (Ip *et al.* 1992a), we hypothesize that *miR-9a* might directly target *rho*. We used TargetScan (Agarwal *et al.* 2018) and SeedVicious (Marco 2018) to computationally identify the presence of two potential *miR-9a* binding sites in the *D. melanogaster rho* 3’UTR (Fig. 1). *rho* has 2 alternatively polyadenylated transcripts (based on the most recent gene annotation in FlyBase), and the predicted *miR-9a* binding sites are both located in the common 3’UTR region. In addition, we used SeedVicious (Marco 2018) to search for *miR-9a* binding sites in Rho orthologs in beetle (*Tribolium castaneum*), worm (*Caenorhabditis elegans*), zebrafish (*Danio rerio*), mouse (*Mus musculus*) and human, and the nonmodel organisms mosquito (*Anopheles gambie*), butterfly (*Heliconius melpomene*) and mite (*Tetranychus urticae*) (Table 1). This analysis shows that *miR-9* family members have predicted binding sites on several rho orthologs. Evidence of conserved miRNA target sites in homologous genes is often an indicator of functional significance (Grün *et al.* 2005; Friedman *et al.* 2009).

**Table 1.** miR-9 binding sites on orthologs of the drosophila Rhomboid.

Organism	Transcript	microRNA	Position on 3’UTR	Site type
*Drosophila melanogaster*	rho-RA/RB	dme-miR-9a/b/c-5p	340	8mer
			1,075	7_A1
*Tribolium castaneum*	TC034044	tca-miR-9b-5p	416	7_m8
		tca-miR-9a/e/c-5p	188	8mer
			417	8mer
*Anopheles gambiae*	AGAP005058 RA/RB	aga-miR-9a/b/c	405	7_A1
			904	8mer
			3,197	8mer
*Heliconius melpomene*	HMEL008701-RA	hme-miR-9b	710	8mer
		hme-miR-9a	1,561	8mer
*Tetranychus urticae*	tetur14g02680.1	tur-miR-9-5p	138	7_A1
*Caenorhabditis elegans*	rho-1	cel-miR-79-3p	54	7_m8
*Danio rerio*	Rhbdl3-203	dre-miR-9-5p	464	7_m8
*Mus musculus*	Rhbdl3-201	mmu-miR-9-5p	1,046	7_m8
*Homo sapiens*	RHBDL3-201/203	hsa-miR-9-3p	2,988	7_A1

**Fig. 2.**
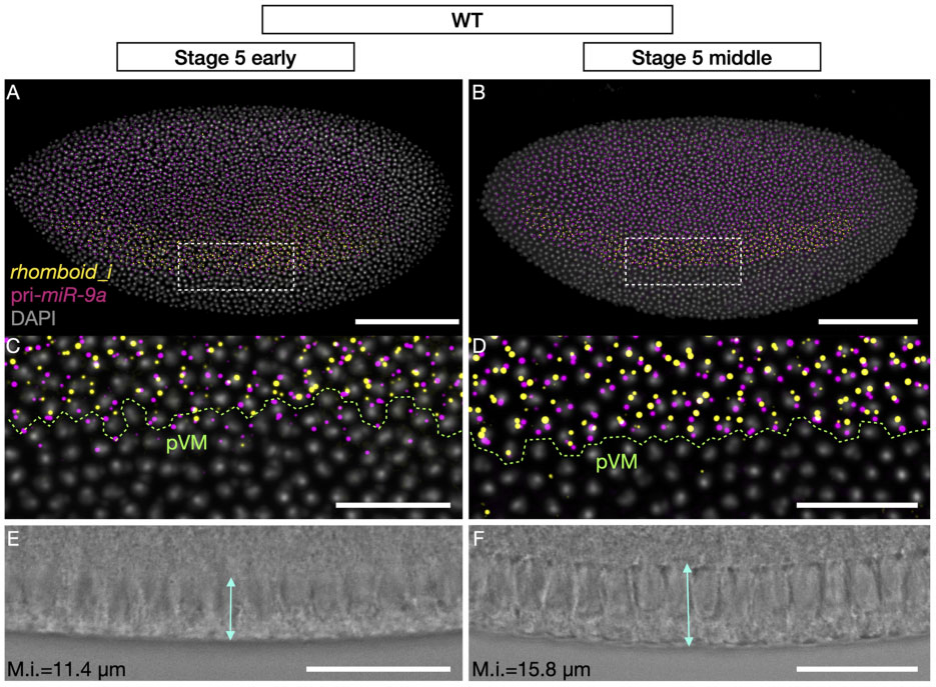
*rhomboid* and *miR-9a* are co-expressed in the neurogenic ectoderm. A) Early and B) middle stage 5 *Drosophila melanogaster* embryos stained with probes against *rhomboid* intron (yellow) and the primary transcript of *miR-9a* (magenta). C-D) Closer sections of highlighted areas in a and b, respectively. In green is highlighted the presumptive ventral midline, which separates mesoderm and ectoderm (pVM). E-F) Brightfields of ventral borders of the embryos in a and b showing membrane introgression (M.i.). Scale bars: 100 μm (A-B), 25 μm (C-F).

We then employed nascent transcript smFISH to precisely establish the overlap in expression domains of *rho* and the primary transcript of *miR-9a* (pri-*mir-9a*). To identify cells that are actively transcribing *rho*, we designed probes against the first intron of *rho* to detect active transcription sites (TS). As mature miRNAs are too short to be detected via smFISH, we designed probes against ∼1kb of sequence flanking the *mir-9a* hairpin to detect the larger primary transcript. Using multiplex smiFISH, we were able to identify cells that are transcribing both *rho* and *mir-9a* at the same time (Fig. 2). As *rho* transcription is activated by Dorsal during embryonic stage 5 (pre-gastrulating embryo) we have focused our imaging on this specific embryonic stage. As expected, *rho*-expressing cells are contained entirely within the *mir-9a* expression domain (Fig. 2, a and b). Since it has been widely observed that gene expression patterns are highly dynamic during stage 5 (Reeves *et al.* 2012), we measured membrane introgression to distinguish between stage 5 sub-stages. We find that both *rho* and *mir-9a* expression pattern become more refined at the ventral edge of their expression domain as stage 5 proceeds (Fig. 2, c–f). Interestingly, while *rho*-expressing cells are generally also expressing *mir-9a*, there are many cells precisely at the ventral edge of the neuroectoderm that are expressing only *mir-9a* (Fig. 2c). As stage 5 progresses, the expression patterns of the two genes become more defined and the ventral expression border of both *rho* and *mir-9a* marks a clear boundary between neurogenic ectoderm and presumptive mesoderm (Fig. 2d). It is therefore likely that the 2 genes respond differently to the Dorsal gradient and to the mesodermal repressor *snail*, which has been shown to repress both *mir-9a* and *rho* in the mesoderm (Hemavathy *et al.* 2004; Fu *et al.* 2014). Taken together, the co-expression of *rho* and *mir-9a* and presence of conserved *miR-9a* target sites suggest that *miR-9a* is a strong candidate to target *rho* mRNA during embryogenesis, and that this role may be evolutionally conserved.

### Increased *rhomboid* mRNA copy number in *miR-9a*^E39^ mutants

**Fig. 3.**
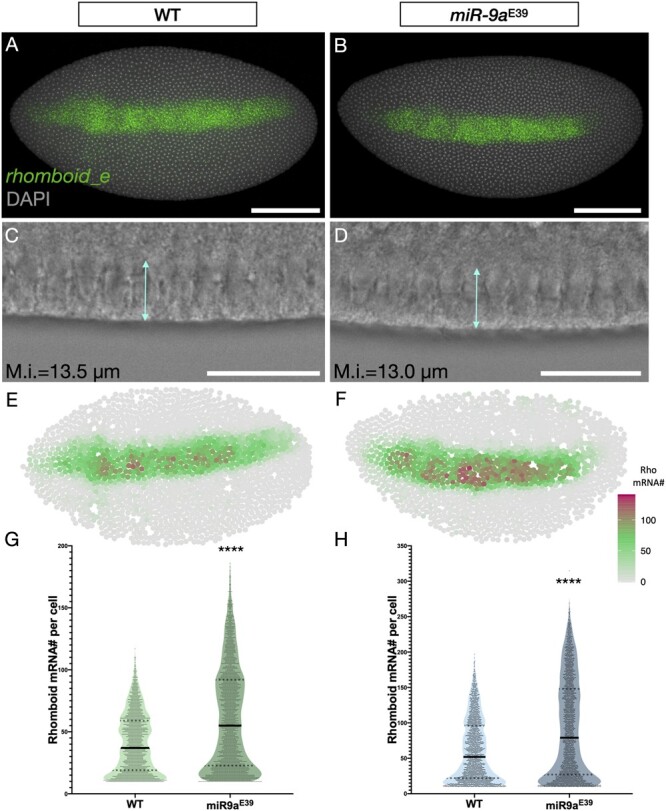
*rhomboid* mRNA number per cell is higher in *miR-9a*^E39^ embryos. A) WT and B) *miR-9a*^E39^ middle stage 5 embryos stained with a probe set against *Rhomboid* transcripts. C-D) Brightfields of a ventral region from embryos in a and b, respectively showing membrane introgression. E-F) Computational reconstruction after segmentation of the embryos in A and B. The colormap is based on mRNA number per cell with gray being low, green intermediate and purple high. G-H) Two independent quantifications of *rhomboid* mRNA number in single cells in WT and *miR-9a*^E39^ mutant embryos. Each quantification was performed using 3 embryos per genotype. Both *P*-values < 0.0001. Scale bars: 100 μm (A-B), 25 μm (C-D).

Combining high-resolution confocal microscopy with smFISH, immunofluorescence and segmentation allows us to count mRNA molecules in individual cells in *Drosophila* early embryos. We quantified *rho* mRNAs per cell in WT and *mir-9a*^E39^ (described in Li *et al.* 2006) stage 5 embryos (Fig. 3, a and b). To precisely determine the stage of embryonic development, we focused only on stage 5 embryos that have a similar level of membrane introgression. As reported in Fu *et al.* (2014) the *rho* expression pattern is not spatially or temporally different in *miR-9a*^E39^ mutant embryos. We imaged and quantified expression in six embryos per genotype, inspected many more and never saw abnormal *rho* expression patterns. Nevertheless, when we performed single-cell segmentation and quantification, differences started to emerge (see Fig. 3, e and f). The data show that the 2 embryos have a spatially equivalent *rho* expression pattern, but the number of mRNAs per cell is higher in *miR-9a*^E39^ mutant embryos. To further support this observation, we performed two independent smFISH experiments using different fluorophores (Fig. 3, g and h), with 3 embryos per genotype. The number of cells that have low or no detected *rho* expression varies from embryo to embryo, likely due to stochastic leaky transcription or false positive detection and counting. After excluding cells with fewer than 10 counted *rho* mRNAs, we found that in both experiments, *miR-9a*^E39^ mutants possess a higher number of *rho* mRNA per cell.

**Fig. 4.**
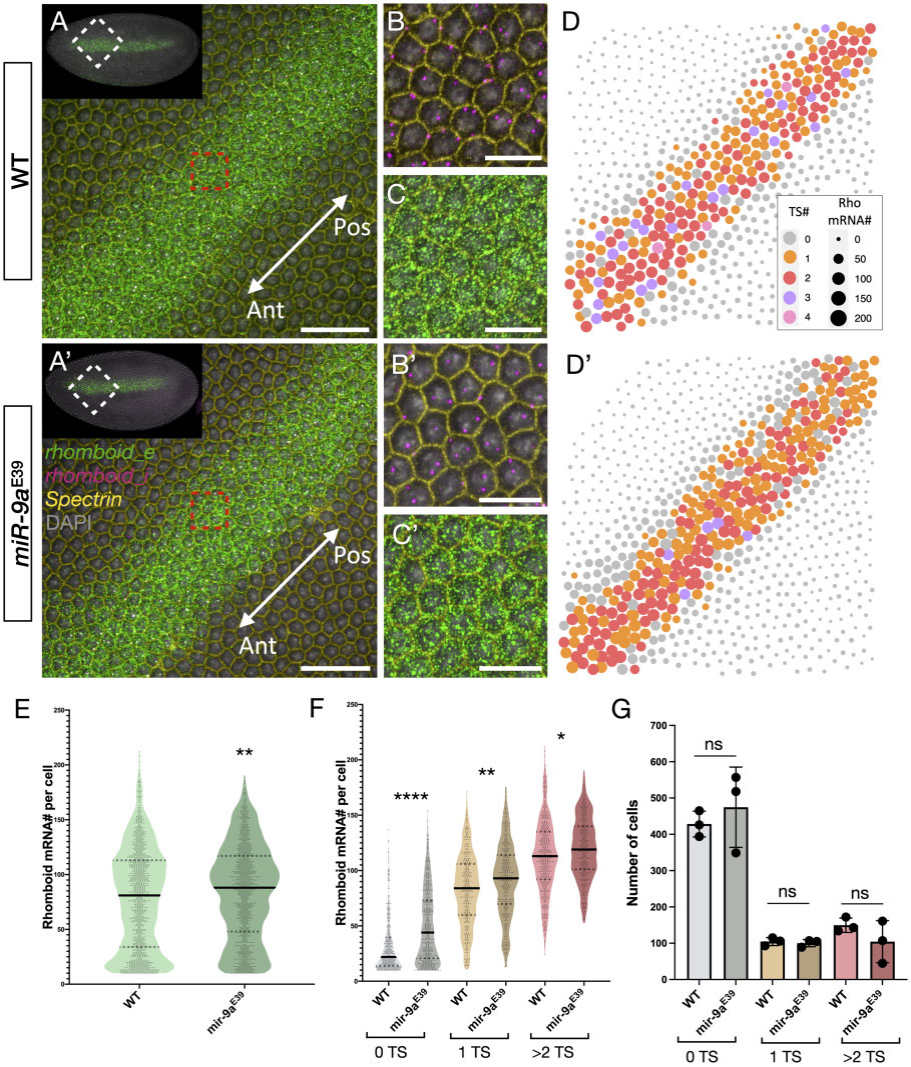
Detection and quantification of *rhomboid* transcription sites in single cells. Central region of A) WT and A') *miR-9a*^E39^ embryos, respectively. Orientation is indicated by the white arrow (ant: anterior embryonic region, pos: posterior embryonic region). B-B') Zoom from red area highlighted in A and A', respectively showing staining against *rhomboid* intron (*rhomboid_i*, magenta), Spectrin to mark cellular membrane (yellow) and DAPI (gray). C-C') Zoom from red area highlighted in A and A', respectively showing staining against *rhomboid* exon (*rhomboid_e,* green), Spectrin and DAPI. D-D') Computational reconstructions of the images in A and A', respectively. Each dot corresponds to a segmented cell. The size of the dot corresponds to the number of *rhomboid* mRNAs detected with *rhomboid_e*, while the color corresponds to the number of detected transcription sites with *rhomboid_i*. E) Comparison between WT and *miR-9a*^E39^*rhomboid* mRNA number per cell. *P*-value = 0.0014. F) Quantified cells are grouped depending on how many alleles are actively transcribing the *rhomboid* locus: gray = 0 alleles active (*P*-value < 0.0001), orange = 1 allele active (*P*-value = 0.0021), red = 2 or more alleles active (*P*-value = 0.0259). G) Bar plot reporting the number of segmented cells belonging to each transcription site group. Colors as in (F). Scale bars: 100 μm (A-A'), 25 μm (B-B', C-C').

To further characterize the difference in *rho* mRNA number in single cells, we simultaneously quantified *rho* transcript sites (TSs) and mature mRNA molecules (Fig. 4), using the intronic probes used in Fig. 2 with *rho* intronic probes used in Fig. 3 targeting the mature *rho* transcripts. We segmented and quantified *rho* TS number per cell (maximum 2 per cell before replication and 4 per cell following). As the higher magnification does not permit imaging of entire embryos, we focused on the central region of the *rho* expressing stripe, again in stage 5 embryos with a similar membrane introgression (Fig. 4, a–c and a’–c’). The comparison of *rho* mRNA distribution between WT and *miR-9a*^E39^ embryos again shows that *miR-9a*^E39^ embryos have higher numbers of *rho* mRNAs per cell (Fig. 4e). The detection and quantification of *rho* TSs allowed us to distinguish between cells that are differentially transcribing *rho*, and thus subgroup them in 3 classes: cells with no TSs, cells with 1 TS and cells with 2 (or more) TSs. In Fig. 4f, we reported that cells with a higher number of TSs also show an increased number of *rho* mRNAs, and for each group of cells, *miR-9a*^E39^ embryos have a generally higher number of transcripts for WT embryos. This becomes particularly evident for cells that are not transcribing *rho* at the moment the embryo was fixed. It is important to note that very few cells have 3 or 4 TSs (<10 per image over ∼700 segmented cells). These may represent cells following DNA replication, or errors in the segmentation process. We are confident that these small numbers do not significantly affect our analysis and we did not observe a change in the number of cells with no TSs, 1 TS or 2 (or more) TSs between the two genotypes (Fig. 4g).

### *miR-9a* does not affect cell-to-cell variation in *rhomboid* mRNA number

**Fig. 5.**
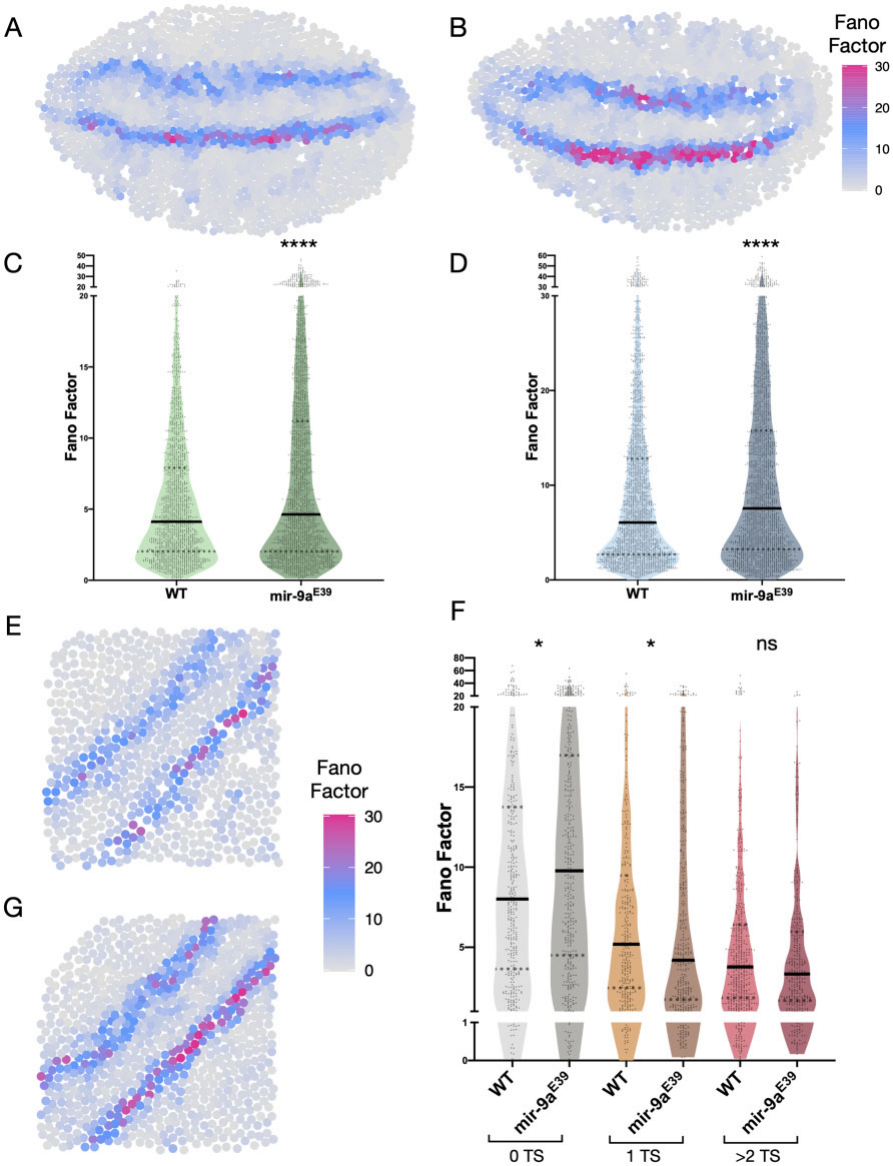
Fano factor quantification and comparison between WT and *miR-9a*^E39^ mutant embryos. Computational reconstruction of Fano factor distribution calculated in neighbor clusters in A) WT and B) *miR-9a*^E39^ stage 5 embryos. These 2 embryos are the same reported in Fig. 3 E and F, respectively. C-D) Comparison between Fano factor in WT and *miR-9a*^E39^ embryos in 2 independent experiments (*n* = 3 embryos each). *P*-value < 0.0001 in both graphs. E-G) Graphical reconstruction of Fano factor distribution calculated in neighbor cells clusters in a WT and *miR-9a*^E39^ embryos, corresponding to Fig. 4, A-A', respectively. F) Cells are sub-grouped depending on their transcription sites number. *P*-values = 0.0147 (0 TS) and 0.0123 (1 TS), ns, nonsignificant.

MicroRNAs are frequently found to have subtle effects on gene expression, acting as buffering factors against intrinsic and extrinsic noise. We, therefore, investigated whether *miR-9a* might not only affect the number of *rho* transcripts per cell, but also cell-to-cell variability in the number of mature mRNAs present. To quantify these effects, we identified the immediate cell neighbors of each segmented cell, and then calculated how variable the *rho* mRNA number per cell is amongst the identified neighbors. As variance scales with mean, areas with high variance do not necessarily correspond to areas in which the cell-to-cell variability is intrinsically higher. Other statistical parameters that have been widely used in order to describe cell-to-cell variability are the coefficient of variation (CV) and the Fano factor (FF) (Munsky *et al.* 2012; Foreman and Wollman 2020). FF is defined as variance/mean while CV as standard deviation/mean. Thus, both measures are mean-normalized. CV is a unitless parameter and has been used to compare cell-to-cell variability between mRNAs or protein levels resulting from the expression of different genes (Foreman and Wollman 2020). On the other hand, FF has a dimension and has been used to measure how the observed data are dispersed from a Poisson distribution (Thattai and Van Oudenaarden 2001; Hortsch and Kremling 2018). As we are comparing measurements relative to the same gene between 2 genotypes, we calculated the FFs for the *rho* mRNA counts reported in Figs. 3 and 4 (see Fig. 5). We observe that the FF is marginally higher in *miR-9a*^E39^ mutants compared to WT, and we posit that it is significantly different because of the very high number of observations, while the effect size is indeed small. Closer inspection shows that the FF is higher in *miR-9a*^E39^ mutants only in the group of cells with no transcription sites, as might be expected, while groups of cells that have a single TS and 2 or more TSs have higher FF in the WT. We speculate that the *miR-9a* buffering action on *rho* mRNA number per cell becomes more evident and/or necessary in quiescent cells that are not actively transcribing *rho*.

### Rho is over-expressed in *miR-9a*^E39^ mutants during embryonic stages 5 and 6

**Fig. 6.**
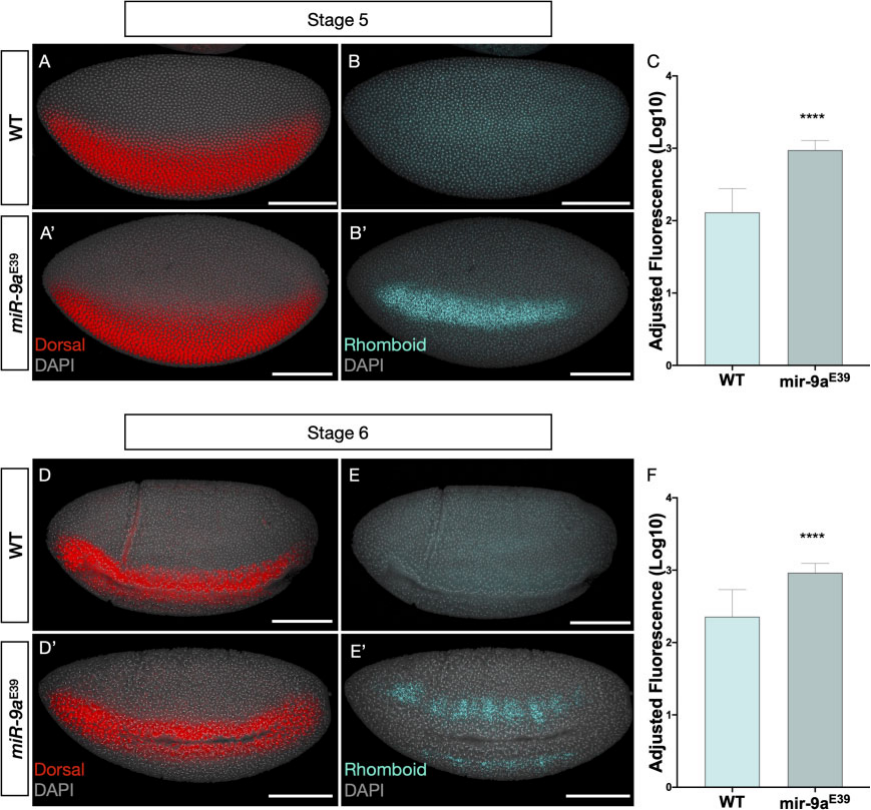
Rhomboid protein is over-expressed in *miR-9a*^E39^ embryos during stages 5 and 6. A-B) Stage 5 WT and a’, b’) *miR-9a*^E39^ embryos, respectively, stained against Dorsal (red) and Rhomboid (cyan). C) Adjusted fluorescence levels from Rhomboid staining in stage 5 embryos (*n* = 3 per genotype). In each embryo 15 areas equally distributed along the Dorsal expression border were quantified. Measurements are reported in Log10 scale. *P*-value < 0.0001. D-E-D'-E') Stage 6 WT and *miR-9a*^E39^ embryos, respectively, stained against Dorsal (red) and Rhomboid (cyan). F) Adjusted fluorescence levels from Rhomboid staining in stage 6 embryos (*n* = 3 per genotype). Quantified as in C). *P*-value < 0.0001. Scale bars: 100 μm in all panels.

As a change in mRNA levels does not necessarily linearly corelate with the change in accumulation of the encoded protein (Koussounadis *et al.* 2015), we compared Rho protein levels between WT and *miR-9a*^E39^ embryos. It has been reported that Rho protein expression is detectable from the embryonic stages 10–11 in WT animals, despite *rho* mRNA being transcribed much earlier during stage 5 (Llimargas and Casanova 1999). However, we find that during stage 5, Rho protein was detectable in *miR-9a*^E39^ embryos. In Fig. 6, we show Rho staining in stage 5 and stage 6 WT and *miR-9a*^E39^ embryos with relative quantifications. Anti-Dorsal antibody was used to provide a further control on the quality of the staining and to orient the embryos. Fluorescence measurements were performed in FIJI by randomly selecting 15 areas per embryo (5 in the anterior, 5 in the central, and 5 in the posterior regions). Quantifications are shown in Fig. 6 (panels C and F for stages 5 and 6, respectively) clearly show that Rho levels are significantly higher (*P*-value < 0.0001 in both cases) in *miR-9a*^E39^ mutants.

## Discussion

*rho* has been one of the most studied Dorsal target genes. Its expression becomes restricted to the neurogenic ectoderm in a precisely orchestrated manner: the low nuclear levels of Dorsal in the dorsal ectoderm do not support *rho* activation, while *snail* represses its transcription in the mesoderm (Ip *et al.* 1992b; Hong *et al.* 2008). *rho* has not been previously studied as a direct target of miRNA regulation, but the combined effect of mutations in *miR-1* and *miR-9a* on *rho* mRNA distribution motivated our investigation into *rho* regulation by miRNAs (Fu *et al.* 2014). We found that the per cell copy number of *rho* mRNA is significantly higher in *miR-9a*^E39^ mutant embryos (Figs. 3 and 4), suggesting *miR-9a* affects *rho* mRNA stability or degradation. Further work is required to determine if this is a direct or indirect effect. We could not find a clear role for *miR-9a* in stabilizing cell-to-cell variability of either the number of *rho* mRNA transcription sites or mRNA molecules (Fig. 5). Nevertheless, when we distinguish between cells that are and are not actively transcribing *rho*, we find that the FF of cells with no transcription sites was significantly higher in *miR-9a*^E39^ mutants. This leads us to suggest that, in WT animals, *rho* mRNA is rapidly degraded when transcription stops, whereas this degradation is less efficient when *miR-9a* is removed, and cell heterogeneity consequently increases. To our knowledge, this is the first study in which mRNA copy number was compared in different genotypes using single-cell quantitative microscopy to uncover miRNA regulatory roles on target gene expression.

It has been shown that protein levels are usually more stable than mRNA levels (Perl *et al.* 2017). The *miR-9a* regulatory effect on Rho protein accumulation might therefore be more evident than the one we observed on the mRNA as it better reflects the integrated activity over time. Rho is a transmembrane protease localized in the Golgi. While Fu *et al.* reported *rho* mRNA patterns in double *miR-9a*/*miR-1* mutants (Fu *et al.* 2014), no information on the protein pattern was previously available. We observed dramatic differences in timing and level of Rho protein accumulation when comparing WT and *miR-9a*^E39^ embryos. In the WT, Rho was only detectable from stage ∼10, whereas in *miR-9a*^E39^ embryos it was clearly present from stage 5, the same stage when we see the initiation of *rho* transcription. The early accumulation of Rho protein appears to be inhibited by *miR-9a.* We suggest that the most parsimonious explanation would be direct translational inhibition by *miR-9a* which is diminished as a certain level of *rho* mRNA is reached, or in response to an external signal later in development. A clear demonstration that the predicted *miR-9a* target sites in the *rho* UTR are functional is needed to further support this hypothesis. We also note the possibility that early low levels of Rho protein may be present but are undetectable with current technology.

Previous work on the *miR-9a*/*miR-1* double mutant shows that when *miR-1* is also removed, strong phenotypic defects emerge leading to failure of gastrulation and ventral midline enclosure (Fu *et al.* 2014). This phenotype suggests that these two miRNAs play an important role in germ layer differentiation. Indeed, while a role for *miR-9a* and *miR-1* involvement in dorso-ventral (DV) axis patterning has not been definitively established, their expression patterns indicate they are early targets of DV specification (Sokol and Ambros 2005; Biemar *et al.* 2006). Our current findings provide convincing evidence for a role of *miR-9a* in the DV patterning process during early *Drosophila* embryogenesis. We posit that *miR-9a* regulates *rho* mRNA accumulation and translation, possibly affecting epidermal growth factor receptor (EGFR) signaling and specification of the dorsal and neurogenic ectoderm (Golembo *et al.* 1996; Guichard *et al.* 1999). The role of *miR-1* is less clear as *miR-1* is not expressed in the same region as *rho*, and therefore *miR-1* can affect *rho* expression only indirectly. *miR-1* is involved in muscle development and is exclusively expressed in the mesoderm (Sokol and Ambros 2005). We suggest that the combination of disrupted *miR-1* function in the mesoderm and *miR-9a* function in the neurogenic ectoderm leads to disruption in establishment or maintenance of an organized border between these 2 germ layers, as seen in the double mutants (Fu *et al.* 2014).

To conclude, we have demonstrated a new function for *miR-9a* during early *Drosophila* embryogenesis. We have observed that *miR-9a* affects both *rho* mRNA copy number per cell (possibly by degradation) and *rho* protein levels. Our findings also show the importance of single-cell quantification when studying the effects of miRNA regulation on target genes. As miRNAs act as weak modulators of gene expression, single-cell quantitative approaches can reveal previously unknown effects on mRNA and protein regulation by miRNAs. This work and the methods described can be easily applied to many other miRNA-target gene networks to allow new insights into miRNA function during development.

## Data availability

Strains and plasmids are available upon request. The authors affirm that all data necessary for confirming the conclusions of the article are present within the article, figures, and tables.

Supplemental material is available at *G3* online.

## Supplementary Material

jkac026_Supplementary_Table_S1

jkac026_Supplementary_Table_S2

jkac026_Supplementary_Table_S3

## References

[jkac026-B1] Agarwal V , Bell GW, Nam JW, Bartel DP. Predicting effective microRNA target sites in mammalian mRNAs. Elife. 2015;4:e05005. 10.7554/eLife.05005 PMC4532895 26267216

[jkac026-B2] Agarwal V , Subtelny AO, Thiru P, Ulitsky I, Bartel DP. Predicting microRNA targeting efficacy in Drosophila. Genome Biol. 2018;19(1):152. 30286781 10.1186/s13059-018-1504-3 PMC6172730

[jkac026-B3] Alvarez-Saavedra E , Horvitz HR. Many families of *C. elegans* MicroRNAs are not essential for development or viability. Curr Biol. 2010;20(4):367–373. 20096582 10.1016/j.cub.2009.12.051 PMC2844791

[jkac026-B4] Bang AG , Kintner C. Rhomboid and star facilitate presentation and processing of the Drosophila TGF-α homolog Spitz. Genes Dev. 2000;14(2):177–186. 10652272 PMC316351

[jkac026-B5] Bartel DP. MicroRNAs: genomics, biogenesis, mechanism, and function. Cell. 2004;116(2):281–297. 14744438 10.1016/s0092-8674(04)00045-5

[jkac026-B6] Bejarano F , Smibert P, Lai EC. miR-9a prevents apoptosis during wing development by repressing Drosophila LIM-only. Dev Biol. 2010;338(1):63–73. 19944676 10.1016/j.ydbio.2009.11.025 PMC2812678

[jkac026-B7] Biemar F , Nix DA, Piel J, Peterson B, Ronshaugen M, Sementchenko V, Bell I, Manak JR, Levine MS. Comprehensive identification of Drosophila dorsal-ventral patterning genes using a whole-genome tiling array. Proc Natl Acad Sci USA. 2006;103(34):12763–12768. 16908844 10.1073/pnas.0604484103 PMC1636694

[jkac026-B8] Bier E , Jan LY, Jan YN. rhomboid, a gene required for dorsoventral axis establishment and peripheral nervous system development in *Drosophila melanogaster*. Genes Dev. 1990;4(2):190–203. 2110920 10.1101/gad.4.2.190

[jkac026-B9] Bonev B , Pisco A, Papalopulu N. MicroRNA-9 reveals regional diversity of neural progenitors along the anterior-posterior axis. Dev Cell. 2011;20(1):19–32. 21238922 10.1016/j.devcel.2010.11.018 PMC3361082

[jkac026-B10] Bonev B , Stanley P, Papalopulu N. MicroRNA-9 modulates Hes1 ultradian oscillations by forming a double-negative feedback loop. Cell Rep. 2012;2(1):10–18. 22840391 10.1016/j.celrep.2012.05.017 PMC4103481

[jkac026-B11] Cassidy JJ , Jha AR, Posadas DM, Giri R, Venken KJT, Ji J, Jiang H, Bellen HJ, White KP, Carthew RW, et al MiR-9a minimizes the phenotypic impact of genomic diversity by buffering a transcription factor. Cell. 2013;155(7):1556–1567. 24360277 10.1016/j.cell.2013.10.057 PMC3891883

[jkac026-B12] Chen X , Yang F, Zhang T, Wang W, Xi W, Li Y, Zhang D, Huo Y, Zhang J, Yang A, et al MiR-9 promotes tumorigenesis and angiogenesis and is activated by MYC and OCT4 in human glioma. J Exp Clin Cancer Res. 2019a;38(1):99. 30795814 10.1186/s13046-019-1078-2 PMC6385476

[jkac026-B13] Chen Y , Shen Y, Lin P, Tong D, Zhao Y, Allesina S, Shen X, Wu C-I. Gene regulatory network stabilized by pervasive weak repressions: microRNA functions revealed by the May-Wigner theory. Natl Sci Rev. 2019b;6(6):1176–1188. 34691996 10.1093/nsr/nwz076 PMC8291590

[jkac026-B14] Chen Y-W , Song S, Weng R, Verma P, Kugler J-M, Buescher M, Rouam S, Cohen SM. Systematic study of Drosophila MicroRNA functions using a collection of targeted knockout mutations. Dev Cell. 2014;31(6):784–800. 25535920 10.1016/j.devcel.2014.11.029

[jkac026-B15] Coolen M , Katz S, Bally-Cuif L. miR-9: a versatile regulator of neurogenesis. Front Cell Neurosci. 2013;7:220. 24312010 10.3389/fncel.2013.00220 PMC3834235

[jkac026-B16] Coolen M , Thieffry D, Drivenes Ø, Becker TS, Bally-Cuif L. MiR-9 controls the timing of neurogenesis through the direct inhibition of antagonistic factors. Dev Cell. 2012;22(5):1052–1064. 22595676 10.1016/j.devcel.2012.03.003

[jkac026-B17] Ferretti E , De Smaele E, Po A, Di Marcotullio L, Tosi E, Espinola MSB, Di Rocco C, Riccardi R, Giangaspero F, Farcomeni A, et al MicroRNA profiling in human medulloblastoma. Int J Cancer. 2009;124(3):568–577. 18973228 10.1002/ijc.23948

[jkac026-B18] Flynt AS , Thatcher EJ, Burkewitz K, Li N, Liu Y, Patton JG. miR-8 microRNAs regulate the response to osmotic stress in zebrafish embryos. J Cell Biol. 2009;185(1):115–127. 19332888 10.1083/jcb.200807026 PMC2700511

[jkac026-B19] Foreman R , Wollman R. Mammalian gene expression variability is explained by underlying cell state. Mol Syst Biol. 2020;16(2):e9146. 32043799 10.15252/msb.20199146 PMC7011657

[jkac026-B20] Freeman M. The rhomboid-like superfamily: molecular mechanisms and biological roles. Annu Rev Cell Dev Biol. 2014;30:235–254. 25062361 10.1146/annurev-cellbio-100913-012944

[jkac026-B21] Friedman RC , Farh KKH, Burge CB, Bartel DP. Most mammalian mRNAs are conserved targets of microRNAs. Genome Res. 2009;19(1):92–105. 18955434 10.1101/gr.082701.108 PMC2612969

[jkac026-B22] Fu S , Nien CY, Liang HL, Rushlow C. Co-activation of microRNAs by Zelda is essential for early drosophila development. Development. 2014;141(10):2108–2118. 24764079 10.1242/dev.108118 PMC4011091

[jkac026-B23] Gallicchio L , Griffiths-Jones S, Ronshaugen M. Single-cell visualization of miR-9a and Senseless co-expression during *Drosophila melanogaster* embryonic and larval peripheral nervous system development. G3 (Bethesda) 2021;11:jkaa010. 33561238 10.1093/g3journal/jkaa010 PMC7849905

[jkac026-B24] Golembo M , Raz E, Shilo BZ. The Drosophila embryonic midline is the site of Spitz processing, and induces activation of the EGF receptor in the ventral ectoderm. Development. 1996;122(11):3363–3370. 8951053 10.1242/dev.122.11.3363

[jkac026-B25] Grün D , Wang YL, Langenberger D, Gunsalus KC, Rajewsky N. MicroRNA target predictions across seven drosophilo species and comparison to mammalian targets. PLoS Comput. Biol. 2005;1:e13. 16103902 10.1371/journal.pcbi.0010013 PMC1183519

[jkac026-B26] Guichard A , Biehs B, Sturtevant MA, Wickline L, Chacko J, Howard K, Bier E. Rhomboid and Star interact synergistically to promote EGFR/MAPK signaling during Drosophila wing vein development. Development. 1999;126(12):2663–2676. 10331978 10.1242/dev.126.12.2663

[jkac026-B27] He L , Zhang L, Wang M, Wang W. miR-9 functions as a tumor inhibitor of cell proliferation in epithelial ovarian cancer through targeting the SDF-1/CXCR4 pathway. Exp Ther Med. 2017;13(4):1203–1208. 28413458 10.3892/etm.2017.4118 PMC5377313

[jkac026-B28] Hemavathy K , Hu X, Ashraf SI, Small SJ, Ip YT. The repressor function of snail is required for Drosophila gastrulation and is not replaceable by Escargot or Worniu. Dev Biol. 2004;269(2):411–420. 15110709 10.1016/j.ydbio.2004.01.029

[jkac026-B29] Hong JW , Hendrix DA, Papatsenko D, Levine MS. How the Dorsal gradient works: insights from postgenome technologies. Proc Natl Acad Sci USA. 2008;105(51):20072–20076. 19104040 10.1073/pnas.0806476105 PMC2629255

[jkac026-B30] Hortsch SK , Kremling A. Adjusting noise in the genetic toggle switch through stochastic circuit design. IFAC. 2018;51(19):68–71.

[jkac026-B31] Ip YT , Park RE, Kosman D, Bier E, Levine M. The dorsal gradient morphogen regulates stripes of rhomboid expression in the presumptive neuroectoderm of the Drosophila embryo. Genes Dev. 1992a;6(9):1728–1739. 1325394 10.1101/gad.6.9.1728

[jkac026-B32] Ip YT , Park RE, Kosman D, Yazdanbakhsh K, Levine M. Dorsal-twist interactions establish snail expression in the presumptive mesoderm of the Drosophila embryo. Genes Dev. 1992b;6(8):1518–1530. 1644293 10.1101/gad.6.8.1518

[jkac026-B33] Kennell JA , Cadigan KM, Shakhmantsir I, Waldron EJ. The microRNA miR-8 is a positive regulator of pigmentation and eclosion in Drosophila. Dev Dyn. 2012;241(1):161–168. 22174085 10.1002/dvdy.23705 PMC3243916

[jkac026-B34] Khafaei M , Rezaie E, Mohammadi A, Shahnazi Gerdehsang P, Ghavidel S, Kadkhoda S, Zorrieh Zahra A, Forouzanfar N, Arabameri H, Tavallaie M, et al miR-9: from function to therapeutic potential in cancer. J Cell Physiol. 2019;234(9):14651–14665. 10.1002/jcp.28210 30693512

[jkac026-B35] Kim TM , Huang W, Park R, Park PJ, Johnson MD. A developmental taxonomy of glioblastoma defined and maintained by microRNAs. Cancer Res. 2011;71(9):3387–3399. 21385897 10.1158/0008-5472.CAN-10-4117 PMC3085663

[jkac026-B36] Koussounadis A , Langdon SP, Um IH, Harrison DJ, Smith VA. Relationship between differentially expressed mRNA and mRNA-protein correlations in a xenograft model system. Sci Rep. 2015;5:1–13. 10.1038/srep10775 PMC4459080 26053859

[jkac026-B37] Kozomara A , Birgaoanu M, Griffiths-Jones S. MiRBase: from microRNA sequences to function. Nucleic Acids Res. 2019;47:155–162. 10.1093/nar/gky1141 PMC6323917 30423142

[jkac026-B38] Leucht C , Stigloher C, Wizenmann A, Klafke R, Folchert A, Bally-Cuif L. MicroRNA-9 directs late organizer activity of the midbrain-hindbrain boundary. Nat Neurosci. 2008;11(6):641–648. 18454145 10.1038/nn.2115

[jkac026-B39] Li X , Cassidy JJ, Reinke CA, Fischboeck S, Carthew RW. A microrna imparts robustness against environmental fluctuation during development. Cell. 2009;137(2):273–282. 19379693 10.1016/j.cell.2009.01.058 PMC2674871

[jkac026-B40] Li Y , Wang F, Lee JA, Gao FB. MicroRNA-9a ensures the precise specification of sensory organ precursors in Drosophila. Genes Dev. 2006;20(20):2793–2805. 17015424 10.1101/gad.1466306 PMC1619947

[jkac026-B41] Linsen SE , Tops BB, Cuppen E. MiRNAs: small changes, widespread effects. Cell Res. 2008;18(12):1157–1159. 19043436 10.1038/cr.2008.311

[jkac026-B42] Liufu Z , Zhao Y, Guo L, Miao G, Xiao J, Lyu Y, Chen Y, Shi S, Tang T, Wu C-I, et al Redundant and incoherent regulations of multiple phenotypes suggest microRNAs’ role in stability control. Genome Res. 2017;27(10):1665–1673. 28904014 10.1101/gr.222505.117 PMC5630030

[jkac026-B43] Llimargas M , Casanova J. EGF signalling regulates cell invagination as well as cell migration during formation of tracheal system in Drosophila. Dev Genes Evol. 1999;209(3):174–179. 10079360 10.1007/s004270050241

[jkac026-B44] Marco A. SeedVicious: analysis of microRNA target and near-target sites. PLoS One. 2018;13(4):e0195532. 29664927 10.1371/journal.pone.0195532 PMC5903666

[jkac026-B45] Matranga C , Tomari Y, Shin C, Bartel DP, Zamore PD. Passenger-strand cleavage facilitates assembly of siRNA into Ago2-containing RNAi enzyme complexes. Cell. 2005;123(4):607–620. 16271386 10.1016/j.cell.2005.08.044

[jkac026-B46] Miska EA , Alvarez-Saavedra E, Abbott AL, Lau NC, Hellman AB, McGonagle SM, Bartel DP, Ambros VR, Horvitz HR. Most *Caenorhabditis elegans* microRNAs are individually not essential for development or viability. PLoS Genet. 2007;3(12):e215. 18085825 10.1371/journal.pgen.0030215 PMC2134938

[jkac026-B47] Morales-Polanco F , Bates C, Lui J, Casson J, Solari CA, Pizzinga M, Forte G, Griffin C, Garner KEL, Burt HE, et al Core Fermentation (CoFe) granules focus coordinated glycolytic mRNA localization and translation to fuel glucose fermentation. iScience. 2021;24(2):102069. 33554071 10.1016/j.isci.2021.102069 PMC7859310

[jkac026-B48] Munsky B , Neuert G, Van Oudenaarden A. Using gene expression noise to understand gene regulation. Science. 2012;336(6078):183–187. 22499939 10.1126/science.1216379 PMC3358231

[jkac026-B49] Ogura Y , Wen FL, Sami MM, Shibata T, Hayashi S. A switch-like activation relay of EGFR-ERK signaling regulates a wave of cellular contractility for epithelial invagination. Dev Cell. 2018;46(2):162–172.e5. 29983336 10.1016/j.devcel.2018.06.004

[jkac026-B50] Okamura K , Hagen JW, Duan H, Tyler DM, Lai EC. The mirtron pathway generates microRNA-class regulatory RNAs in Drosophila. Cell. 2007;130(1):89–100. 17599402 10.1016/j.cell.2007.06.028 PMC2729315

[jkac026-B51] Otaegi G , Pollock A, Hong J, Sun T. MicroRNA miR-9 modifies motor neuron columns by a tuning regulation of FoxP1 levels in developing spinal cords. J Neurosci. 2011;31(3):809–818. 21248104 10.1523/JNEUROSCI.4330-10.2011 PMC3040495

[jkac026-B52] Perl K , Ushakov K, Pozniak Y, Yizhar-Barnea O, Bhonker Y, Shivatzki S, Geiger T, Avraham KB, Shamir R. Reduced changes in protein compared to mRNA levels across non-proliferating tissues. BMC Genomics. 2017;18(1):305. 28420336 10.1186/s12864-017-3683-9 PMC5395847

[jkac026-B53] Peter ME. Targeting of mRNAs by multiple miRNAs: the next step. Oncogene. 2010;29(15):2161–2164. 20190803 10.1038/onc.2010.59

[jkac026-B54] Rand TA , Petersen S, Du F, Wang X. Argonaute2 cleaves the anti-guide strand of siRNA during RISC activation. Cell. 2005;123(4):621–629. 16271385 10.1016/j.cell.2005.10.020

[jkac026-B55] Reeves GT , Trisnadi N, Truong TV, Nahmad M, Katz S, Stathopoulos A. Dorsal-ventral gene expression in the Drosophila embryo reflects the dynamics and precision of the dorsal nuclear gradient. Dev Cell. 2012;22(3):544–557. 22342544 10.1016/j.devcel.2011.12.007 PMC3469262

[jkac026-B56] Reinhart BJ , Slack FJ, Basson M, Pasquinelli AE, Bettinger JC, Rougvie AE, Horvitz HR, Ruvkun G. The 21-nucleotide let-7 RNA regulates developmental timing in *Caenorhabditis elegans*. Nature. 2000;403(6772):901–906. 10706289 10.1038/35002607

[jkac026-B57] Sokol NS , Ambros V. Mesodermally expressed Drosophila microRNA-1 is regulated by Twist and is required in muscles during larval growth. Genes Dev. 2005;19(19):2343–2354. 16166373 10.1101/gad.1356105 PMC1240043

[jkac026-B58] Soto X , Biga V, Kursawe J, Lea R, Doostdar P, Thomas R, Papalopulu N. Dynamic properties of noise and Her6 levels are optimized by miR‐9, allowing the decoding of the Her6 oscillator. EMBO J. 2020;39(12):e103558. 32395844 10.15252/embj.2019103558 PMC7298297

[jkac026-B59] Thattai M , Van Oudenaarden A. Intrinsic noise in gene regulatory networks. Proc Natl Acad Sci USA. 2001;98(15):8614–8619. 11438714 10.1073/pnas.151588598 PMC37484

[jkac026-B60] Trcek T , Lionnet T, Shroff H, Lehmann R. mRNA quantification using single-molecule FISH in Drosophila embryos. Nat Protoc. 2017;12(7):1326–1347. 28594816 10.1038/nprot.2017.030 PMC6668020

[jkac026-B61] Tsanov N , Samacoits A, Chouaib R, Traboulsi A-M, Gostan T, Weber C, Zimmer C, Zibara K, Walter T, Peter M, et al SmiFISH and FISH-quant - a flexible single RNA detection approach with super-resolution capability. Nucleic Acids Res. 2016;44(22):e165. 27599845 10.1093/nar/gkw784 PMC5159540

[jkac026-B62] Urban S , Lee JR, Freeman M. Drosophila Rhomboid-1 defines a family of putative intramembrane serine proteases. Cell. 2001;107(2):173–182. 11672525 10.1016/s0092-8674(01)00525-6

[jkac026-B63] Valencia-Sanchez MA , Liu J, Hannon GJ, Parker R. Control of translation and mRNA degradation by miRNAs and siRNAs. Genes Dev. 2006;20(5):515–524. 16510870 10.1101/gad.1399806

[jkac026-B64] van Rooij E , Sutherland LB, Qi X, Richardson JA, Hill J, Olson EN. Control of stress-dependent cardiac growth and gene expression by a microRNA. Science. 2007;316(5824):575–579. 17379774 10.1126/science.1139089

[jkac026-B65] Xu P , Vernooy SY, Guo M, Hay BA. The Drosophila microRNA mir-14 suppresses cell death and is required for normal fat metabolism. Curr Biol. 2003;13(9):790–795. 12725740 10.1016/s0960-9822(03)00250-1

[jkac026-B66] Zhao C , Sun G, Li S, Shi Y. A feedback regulatory loop involving microRNA-9 and nuclear receptor TLX in neural stem cell fate determination. Nat Struct Mol Biol. 2009;16(4):365–371. 19330006 10.1038/nsmb.1576 PMC2667220

